# Fungal Laccases Degradation of Endocrine Disrupting Compounds

**DOI:** 10.1155/2014/614038

**Published:** 2014-04-15

**Authors:** Gemma Macellaro, Cinzia Pezzella, Paola Cicatiello, Giovanni Sannia, Alessandra Piscitelli

**Affiliations:** Department of Chemical Sciences, University of Naples “Federico II”, Complesso Universitario Monte S. Angelo, Via Cinthia 4, 80126 Naples, Italy

## Abstract

Over the past decades, water pollution by trace organic compounds (ng/L) has become one of the key environmental issues in developed countries. This is the case of the emerging contaminants called endocrine disrupting compounds (EDCs). EDCs are a new class of environmental pollutants able to mimic or antagonize the effects of endogenous hormones, and are recently drawing scientific and public attention. Their widespread presence in the environment solicits the need of their removal from the contaminated sites. One promising approach to face this challenge consists in the use of enzymatic systems able to react with these molecules. Among the possible enzymes, oxidative enzymes are attracting increasing attention because of their versatility, the possibility to produce them on large scale, and to modify their properties. In this study five different EDCs were treated with four different fungal laccases, also in the presence of both synthetic and natural mediators. Mediators significantly increased the efficiency of the enzymatic treatment, promoting the degradation of substrates recalcitrant to laccase oxidation. The laccase showing the best performances was chosen to further investigate its oxidative capabilities against micropollutant mixtures. Improvement of enzyme performances in nonylphenol degradation rate was achieved through immobilization on glass beads.

## 1. Introduction

In the last years assessment and conservation of environmental quality have represented an interesting field of technologic applications. The main problem in industrialized states is represented by a constant and continuous pollution of soil, water-bearing stratum, surface water, and air. This is due to the introduction, in the environment, of toxic and dangerous contaminants for many organisms, including humans. In this context endocrine disrupting chemicals (EDCs) play a significant role. EDCs have been found to disturb the endogenous hormone pathway and interrupt the function of hormone receptors via estrogens-mimicking chemicals, resulting in the alteration of physiological functions, such as reproduction and development of different species, including humans [1]. EDCs are found in many products derived from cosmetic industries and working environment [2]. Many natural chemicals (e.g., phytoestrogens, including genistein and coumestrol), found in human and animal food, can also act as endocrine disruptors [2, 3].

Between 2000 and 2006 the European Commission has contracted diverse studies on the identification and evaluation of this class of substances, and a list of substances potentially endocrine disruptor has been drawn up [4]. Efficient and applicable techniques for removing EDCs in wastewater treatment processes remain a challenge of high environmental and public health significance [5]. One promising approach consists in the use of enzymatic systems able to degrade EDCs into nontoxic or easy to remove products [6]. The promise of phenol oxidases (laccases and tyrosinases) and peroxidases for the elimination of EDCs from aqueous solutions has been established over the last few years and is attracting an increasing attention [7, 8]. Nonetheless, the application of enzymes in continuous systems such as wastewater treatment plants remains a challenge as it is limited by their non-reusability, the instability of their structures, and their sensitivity to harsh process conditions. Many of these undesirable limitations may be overcome by the use of immobilized enzyme. In the immobilized form, enzymes are more robust and more resistant to environmental changes allowing easy recovery and multiple reuses [8].

As a fact, examples referring to treatment of EDCs molecules [9–11], as well as of contaminated synthetic water and municipal wastewater [12], with different fungal peroxidases, laccases, and tyrosinases are present in the recent literature. In all reported cases, estrogenic activities were completely removed. Recent efforts have been focused on the immobilization of biocatalysts in order to tackle this major limitation and to facilitate their possible reuse [8].

Laccases (p-diphenol-dioxygen oxidoreductases; EC 1.10.3.2) are blue multicopper oxidases, catalysing the oxidation of a broad range of xenobiotics concomitantly with the reduction of molecular oxygen to water. This renders them very attractive compared to other enzymatic systems because no additional/expensive cosubstrate or cofactor is required apart from oxygen. These enzymes usually contain four copper ions distributed in three active sites, which are involved in the electron transfer from the substrate (T1 active site) towards oxygen (T2/T3 active sites) [13].

**Figure 1:**
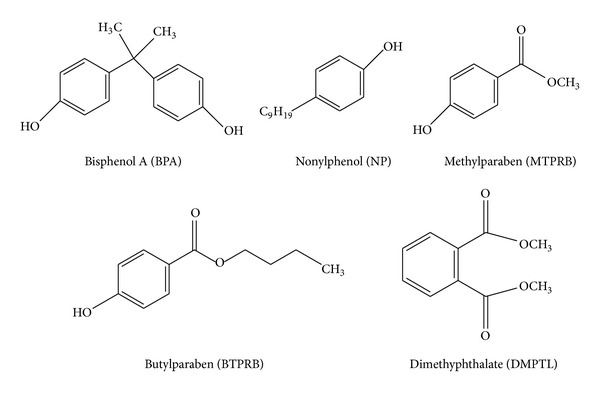
Chemical structure of endocrine disrupting substances used in this study.

In this project, among various chemical classes, the EDCs bisphenol A (BPA), nonylphenol (NP), methylparaben (MTPRB), butylparaben (BTPRB), and dimethylphthalate (DMPTL) (Figure 1) have been selected, based on information about their toxicity, the amount discharged per year, and their commercial availability. BPA is a high production volume chemical used as an intermediate in the fabrication of polycarbonate plastic and epoxy resins [14]. Due to its daily use, high concentrations of BPA are observed in wastewater and in wastewater sludge (0.004–1.36 mg kg^−1^). NP is a mixture of para-, ortho-, and metaisomers; the most prevalent of them is para-NP. It is a viscous, colourless liquid and it is subjected to ethoxylation to give alkylphenol ethoxylates [15]. This compound is very toxic and recalcitrant; thus it shows a high potential to bioconcentrate [16]. Parabensare esters of parahydroxybenzoic acid, widely used as preservatives in food, pharmaceutical, and cosmetic industries to prevent bacterial growth [17, 18]. Phthalates are a group of persistent, high production volume chemicals, used for a variety of products, including personal care products (e.g., perfumes, lotions, and cosmetics), varnish, medical devices, pharmaceuticals, solvents, additives, and insect repellents [19].

Four different fungal laccases were used in this study to set up EDCs enzymatic treatment, also in the presence of both synthetic and natural mediators. Three out of four selected enzymes were high redox potential laccases from* Pleurotus ostreatus*: POXC [20, 21], POXA1b [21, 22] heterologously expressed in the filamentous fungus* Aspergillus niger* [23], 1H6C, a POXA1b variant obtained through random mutagenesis [24] and produced in* A. niger* [23]. Finally, a commercial laccase, the Novoprime Base 268 (Novozymes), was also used for enzymatic treatment. Moreover, considering that in the natural environment pollutant mixtures are common, this study also evaluated the effect of the best performing enzyme, both free and immobilized, towards the presence of pollutants mixtures.

## 2. Materials and Methods

### 2.1. Organism and Culture Conditions

The* P. ostreatus* (Jacq.: Fr.) Kummer (type: Florida) (ATCC number MYA-2306) fungus was maintained through periodic transfer at 4°C on potato dextrose yeast extract (PDY) 24 g/L potato dextrose; 5 g/L yeast extract. Growth was carried out at 28°C in the dark by preinoculating 300 mL of PDY in 500 mL shaken flasks with 6 agar plugs of mycelium grown on solid state on Petri dishes (11 mm diameter). 50 mL of a 5-day-old culture was transferred in 1 L flasks containing 450 mL of PDY broth. Cultures were incubated in the dark at 28°C under agitation (150 rpm).

*A. niger* D15#26 strain [25] was grown in liquid medium (300 mL) containing 70 mM NaNO_3_, 7 mM KCl, 200 mM Na_2_HPO_4_, 2 mM MgSO_4_·7H_2_O, 5% (w/v) glucose, 2 g/L casamino acids, and 5 g/L yeast extract. pH was daily adjusted to 5.0 by adding 1 M citric acid [23].

### 2.2. Enzymes

Laccase POXC [20] was purified from* P. ostreatus* with slight modifications in the purification protocol. After 10 days of culture, the medium was collected and filtered through gauze. 1 mM of the serine protease inhibitor, phenylmethanesulfonyl fluoride (PMSF), was added to the supernatant. Secreted proteins were precipitated from the filtered medium by addition of (NH_4_)_2_SO_4_ up to 80% saturation and loaded on Phenyl Sepharose High Performance 35/100 (GE Healthcare, Milan, Italy). POXC was eluted with a linear gradient of decreasing (NH_4_)_2_SO_4_ concentration from 1 M to 0 M. Fractions corresponding to POXC were pooled, equilibrated in buffer 50 mM sodium phosphate (NaP) pH 6.5, and loaded onto a DEAE Sepharose Fast Flow column (GE Healthcare, Milan, Italy) with a linear gradient from 0 M to 0.5 M NaCl, and fractions corresponding to POXC were pooled and desalted.

POXA1b and 1H6C were heterologously expressed and purified from* A. niger*, as previously described [23].

Laccase Novoprime Base 268 (Novozymes) was dissolved in 50 mM NaP pH 6.5.

### 2.3. Assay of Enzymatic Activity

Laccase activity was assayed at 25°C by monitoring the oxidation of 2,2′-azino-bis(3-ethylbenzothiazoline-6-sulphonic acid) (ABTS) at 420 nm (*ε*_420_ = 36 × 10^3^ M^−1 ^cm^−1^). The assay mixture contained 2 mM ABTS in 100 mM sodium citrate buffer, pH 3.0.

Immobilized enzyme activity was assayed incubating 10 mg of glass beads in 1 mL of 2 mM ABTS in 100 mM sodium citrate buffer (pH 3.0). The activity was determined by measuring the absorbance at 420 nm every 30′′ following the reaction for 2 min. Enzymatic units were expressed as U/g.

### 2.4. Laccase Immobilization on Glass Beads

Glass beads type S (0.4–0.6 mm diameter) were supplied by Silibeads (Sigmund Lindner GmbH, Germany). Beads were pretreated with 1.2 M HNO_3_ at 60°C for 4 hours, extensively washed with water, and then dried at 60°C. Carrier derivatization was performed as follows: 5 g of dry pretreated beads was mixed with 10% APTES (*γ*-aminopropyltriethoxysilane, Sigma-Aldrich) in 50 mL distilled water and incubated at 80°C for 2 h under constant mixing. The suspension was then washed thoroughly with 50 mM NaP buffer pH 6.5 and treated with 2.5% glutaraldehyde for 1 h at room temperature. The activated beads were extensively washed with the overcited buffer and finally incubated for 1 h with a solution of laccase mixture in 50 mM NaP buffer, pH 6.5 at room temperature. Residual active glutaraldehyde was inactivated by 1 h incubation with 100 mM glycine at room temperature. Immobilization yield (Y) was defined as the ratio between laccase activity assayed on the solid biocatalyst and total activity available in the liquid solution at the beginning of the immobilization processes. A yield of 83% was obtained following this procedure.

### 2.5. EDCs Enzymatic Degradation

1 mM stock solution of each EDC (Sigma-Aldrich, Milan, Italy) was prepared in hot water. To improve the solubility of NP and DMPTL in hot water, methanol (0.4% v/v) and Tween 80 (0.1% w/v) were added, respectively. 100 *μ*M of each EDC was incubated for 1 h at 25°C in a reaction mixture containing 1.5 U/mL of purified laccase in 50 mM sodium citrate buffer, pH 5.0; total reaction volume was set to 4 mL. Amounts of EDC were quantified every 30 minutes (*t*_0_, *t*_30′_, *t*_60′_) by reverse-phase HPLC. Enzymatic reaction was stopped by adding 50 *μ*L of hydrochloric acid (HCl) to 500 *μ*L of reaction mixture and centrifuging at 15, 100 g for 15 min at room temperature. 100 *μ*L of the supernatant was analysed by HPLC. Degradation of EDCs mixture was performed in the same condition, using a final concentration of 25 *μ*M of each EDC, but for DMPTL. Thus, the final concentration of EDCs mixture was of 100 *μ*M. Control reactions were always performed in the same conditions without enzyme addition. Mediators used were ABTS, dissolved in sodium citrate buffer 50 mM, pH 5.0, and acetosyringone (AS), dissolved in hot sodium citrate buffer, 50 mM, pH 5.0. Concentrations used for both mediators were 20 *μ*M and 200 *μ*M.

Degradation of EDCs mixture by means of immobilized enzyme was performed in the same conditions, using an amount of beads corresponding to 6 U total in the presence of 20 *μ*M AS.

### 2.6. High-Performance Liquid Chromatography

All EDCs were quantitatively analysed using a C18 column (Grace Vydac, Hesperia, CA, USA) on an HPLC instrument (Agilent Technologies, Italy). The fractions were eluted by using a linear gradient of water-acetonitrile (A solvent 0.1% trifluoroacetic acid in Milli-Q (MQ) water; B solvent 0.07% trifluoroacetic acid, 5% MQ water in acetonitrile) at a flow rate of 1 mL/min. The gradient program for BPA analysis was 0–3 min (acetonitrile 30%), 3–9 min (acetonitrile 30–90%), 9–12 min (acetonitrile 90%), 12-13 min (acetonitrile 90–30%), and 13–15 min (acetonitrile 30%). The eluted sample was monitored by UV absorbance at 227 nm. The retention time for BPA was 6.9 min under these conditions. As regards NP, the applied gradient was 0–3 min (acetonitrile 20%), 3–9 min (acetonitrile 20–90%), 9–12 min (acetonitrile 90%), 12-13 min (acetonitrile 90–20%), and 13–15 min (acetonitrile 20%). The detection wavelength was 277 nm. The retention time for NP was 14.5 min under these conditions. The gradient program for parabens analyses was 0–7 min (acetonitrile 30–70%), 7-8 min (acetonitrile 70–90%), 8–11 min (acetonitrile 90%), 11-12 min (acetonitrile 90–30%), and 12–14 min (acetonitrile 30%). The detection wavelength was 254 nm. Under these conditions the retention times for MTPRB and BTPRB were 5.8 min and 8.8 min, respectively. As regards DMPTL, the applied gradient was the same used for parabens, while the detection wavelength was 274 nm. The retention time for DMPTL was 6.8 min under these conditions.

As far as the EDCs mixtures are concerned, each molecule was analysed with its optimised program.

The peak area on the chromatogram was used to calculate the remaining amount of EDC as a percentage of the initial value.

## 3. Results and Discussion

### 3.1. Endocrine Disruptors Degradation by Enzymes

**Figure 2:**
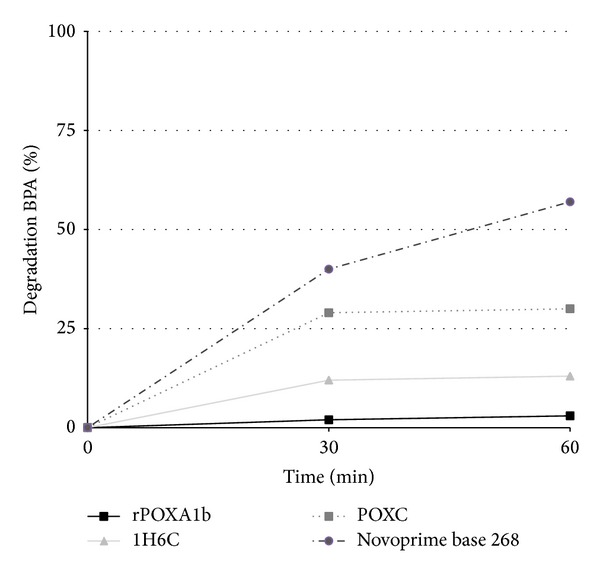
Percentage degradation (%) of BPA by fungal laccases. Reaction conditions: 100 *μ*M BPA, pH 5.0 (50 mM sodium citrate buffer), 25°C, and 1.5 U/mL laccase, with a reaction time of 1 h. All results are averages from two replicate experiments and the standard deviation is less than 10%.

Enzymatic degradation of EDC bisphenol A (BPA), nonylphenol (NP), methylparaben (MTPRB), butylparaben (BTPRB), and dimethylphthalate (DMPTL) was tested in solution at pH 5.0 in the presence of the different selected laccases. Among the EDC molecules, only BPA was degraded by enzymes in the absence of any mediator within the time of incubation analysed (Figure 2). After 1 hour of incubation Novoprime Base 268 was able to degrade 60% of BPA, whereas POXC degradation rate was slower than that obtained by Novoprime 268, reaching 30% of BPA degradation after both 30 minutes and 1 h incubation. Both POXA1b and 1H6C were less efficient, with the latter being more able to degrade BPA, probably thanks to its higher redox potential [23]. The rate of BPA degradation was comparable with that obtained for other laccases in similar conditions. A careful comparison of results present in the recent scientific literature reveals that different strategies have been used to obtain BPA removal, along with different time of reaction and concentration of both enzyme and substrate. Gassara and coworkers [26] reported a rate of BPA degradation of 13% after 2 hours of incubation in the presence of 0.05 U/mL of a laccase from* Phanerochaete chrysosporium*. A purified laccase from* Grifola frondosa* was able to degrade 15% BPA (0.65 mM) in 1 hour [27], whereas a purified laccase from* Phlebia tremellosa* [28] removed around 65% of BPA estrogenic activity after 3 h incubation with 50 U of enzymatic activity. Interesting results were obtained using a purified laccase by* Trametes villosa*, able to totally degrade 2.2 mM BPA after 3 h incubation [29].

### 3.2. EDCs Degradation by Enzymes in the Presence of Mediators

With the aim to enhance laccase efficiencies towards selected EDCs, two different mediators, a synthetic and a natural one, were added to the reaction mix. The selected mediators were ABTS, the first acknowledged laccase mediator [30], and the natural mediator AS, an eco-friendly, easily and economically available mediator [31].

**Figure 3:**
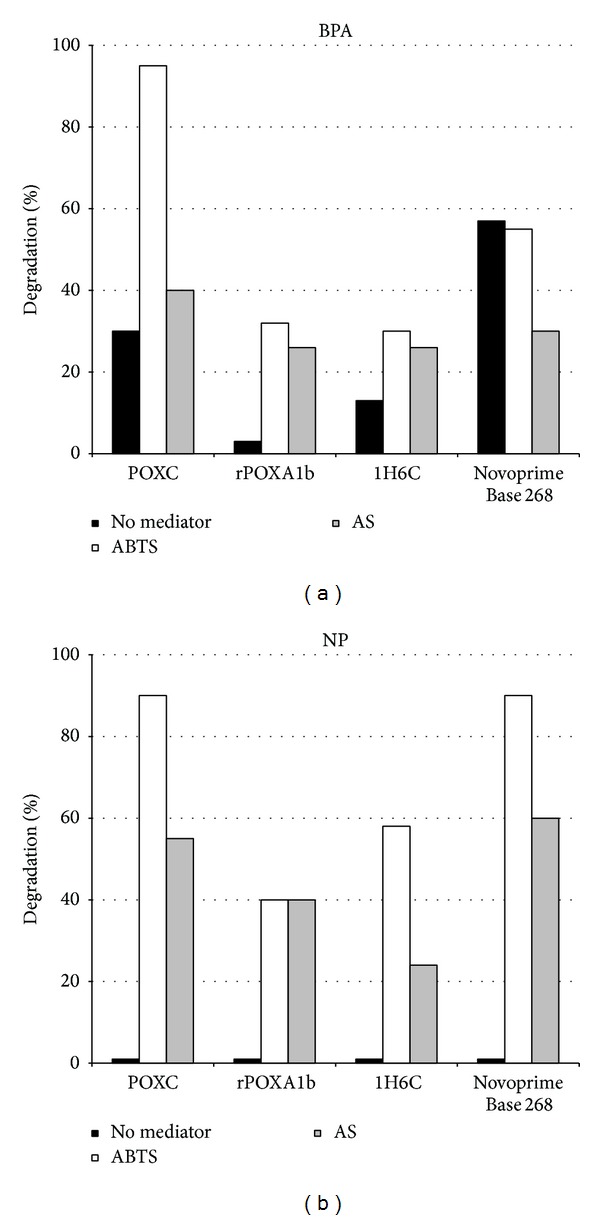
Effect of absence of mediator, 20 *μ*M of ABTS, or AS on the removal of EDCs after a 1 h treatment at pH 5.0 and at a temperature of 25°C with 1.5 U/mL of laccases. (a) BPA; (b) NP. All results are averages from two replicate experiments and the standard deviation is less than 10%.

As it is shown in Figure 3(a), the presence of both mediators enhances laccase performances towards BPA but for Novoprime 268 and ABTS mediator is more effective than AS with all the tested laccases. As a fact, in the presence of ABTS, POXC was able to almost fully degrade BPA (95%) after 1 hour reaction. It is also possible to note that in the presence of both mediators POXA1b and 1H6C showed the same efficiency. Unexpectedly, the presence of mediators did not influence or even decreased Novoprime base 268 efficiency. A similar effect has also been observed for a* Coriolopsis polyzona* laccase towards NP using 1-hydroxybenzotriazole (HBT) as mediator [10].

Also when considering nonylphenol, the presence of both mediators enhances laccase performances, with ABTS being more effective than AS with all tested laccases (Figure 3(b)). In this case, POXC and Novoprime base 268 showed almost the same degradation rate both in the presence of ABTS and AS. On the other hand, POXA1b and 1H6C showed an opposite behaviour. As a fact, in the presence of ABTS, 1H6C was more effective than POXA1b, whereas in the presence of AS, POXA1b proved to be more efficient than its variant. This result seems to indicate that no simple rule regarding redox potential or affinity can be easily drawn, as the whole reaction mechanism is quite complex. The obtained results seem promising if carefully compared with other systems. Indeed, a laccase from the white rot fungus* C. polyzona* was able to eliminate 50% BPA and 66% NP in the presence of 10 *μ*M ABTS as mediator [10]. When the synthetic mediator HBT (200 *μ*M) was used to improve laccase degradation, an enhanced degradation of almost 1.3-fold for both substrates was observed, reaching a degradation of 95% and 80% for BPA and NP, respectively [32].

When the mediator concentration was increased up to 200 *μ*M, AS was revealed to be the best mediator, since all enzymes were able to also degrade methylparaben and butylparaben after 1 h incubation (Table 1). Also in this case, POXC showed the best performances, being able to degrade in 30 minutes 50% and 60% of methyl and butylparaben, respectively (degradation did not improve after 1 h incubation). Among parabens, butylparaben was more susceptible to laccase degradation in the presence of mediators than methylparaben. In the scientific literature are present only few reports regarding paraben degradation by laccases. Mizuno and coworkers [33] demonstrated that both iso-butylparaben and n-butylparaben were almost completely removed (95%) after 2 h of treatment and completely disappeared after 4 h of treatment with 0.5 U/mL of laccase activity in the presence of 2 mM HBT. The only substrate recalcitrant to laccase oxidation in all the tested conditions was dimethylphthalate.

### 3.3. Degradation of EDCs Mixture by Free and Immobilized POXC

**Figure 4:**
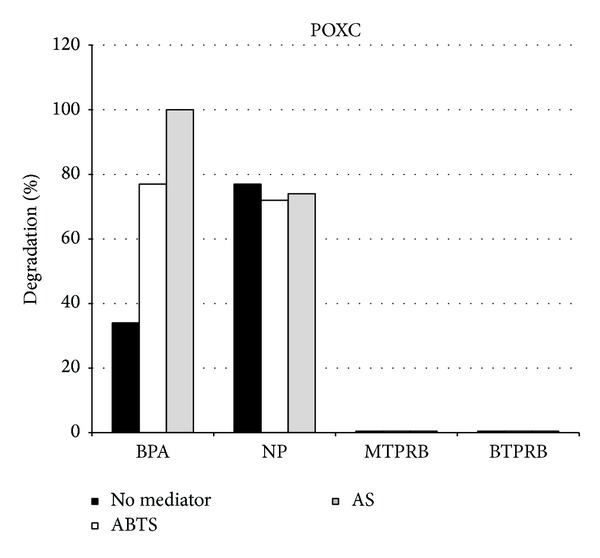
Effect of absence of mediator, 20 *μ*M of ABTS, or AS on the removal of EDCs mixtures by POXC. Reaction conditions: 25 *μ*M of each molecule, pH 5.0 (50 mM sodium citrate buffer), 25°C, and 1.5 U/mL laccase, with a reaction time of 1 h. All results are averages from two replicate experiments and the standard deviation is less than 10%.

POXC, the best performing enzyme, was chosen for further degradation analyses against a mixture of the selected EDCs in a total final concentration of 100 *μ*M. The analyses were conducted in the presence of four out of five substrates. As a fact, DMPTL was not used, considering its recalcitrance to laccase degradation under all the tested conditions. It is worth to note that in the absence of any mediator POXC is able to degrade almost 40% BPA and 80% NP after 1 h incubation, whereas methyl and butylparaben were not degraded (Figure 4). As far as BPA is concerned, a slower degradation rate was observed when BPA concentration was lowered if compared with the degradation observed with high BPA concentration. When mediator was added to the reaction, the efficiency was greatly enhanced, and full disappearance of BPA was observed in the presence of AS. On the other hand, POXC is able to efficiently degrade NP at low concentration also in the absence of mediators, and no increase is observed when mediators are added to the reaction mix. Thus, it may be hypothesized that the enzyme shows a higher affinity towards NP than towards BPA. Parabens at low concentration were not oxidised in the presence of both mediators.

When immobilized POXC was used towards EDCs mix in the presence of AS, NP degradation improved with respect to the free enzyme, reaching the same extent of degradation (80%) within only 15 min, and no further increase was observed. On the other hand, a slightly lower BPA removal (80%) was observed using the immobilized enzyme with respect to the free one. Parabens were not degraded, following the same trend already observed for the soluble counterpart. Control reactions were carried out using the silanized and derivatized carrier (without enzyme) against the mix of EDCs and no adsorption on the carrier was observed. Laccase immobilized on glass beads maintained significant activity during storage at 4°C in 50 mM phosphate buffer pH 6.5. After one month of storage, the retained laccase activity was 100%.

**Figure 5:**
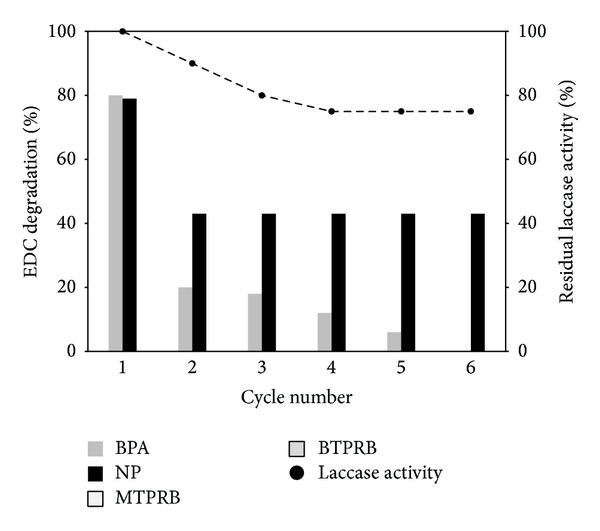
Percentage degradation (%) of BPA and NP by immobilized POXC. Reaction conditions: 6 U_TOT_ versus 25 *μ*M each EDC, pH 5.0 (50 mM sodium citrate buffer), 25°C, and in the presence of 20 *μ*M AS with a reaction time of 1 h. Residual laccase activity is reported as filled black circle. All results are averages from two replicate experiments and the standard deviation is less than 10%.

In order to assess reusability of the immobilized laccase against mixture of EDCs, six successive cycles of batch degradation were performed. After six cycles, there was a 20% drop in laccase activity (Figure 5). As far as EDC removal is concerned, a gradual loss of BPA degradation during six cycles was observed. On the other hand, NP degradation was decreased up to 40% after the first cycle, but no further drop was observed during the following 5 cycles.

## 4. Conclusions

The growing attention accorded to the removal of EDCs from environmental matrices makes oxidative enzymes an attractive candidate in the bioremediation arsenal. Four different laccases were chosen for their interesting characteristics and tested towards EDC molecules. The obtained results have shown that all laccases are able to oxidize different EDCs. In particular, BPA is the only substrate oxidized under all conditions tested. Furthermore, to improve laccase capabilities, mediators were added to reaction mixtures. Among the chosen laccases, POXC was the enzyme with the highest bioremediation capacity under all conditions analysed. Its performance was increased in the presence of both mediators. Interesting results were obtained in the presence of the natural mediator acetosyringone. When used at high concentration, this natural mediator enhanced the bioremediation capacity of POXC determining a rate degradation of 50% of both parabens in 30 minutes. Thus, results herein obtained confirm laccase capabilities [33] to degrade this kind of substrates, very poorly investigated till now. Furthermore, oxidative capabilities of POXC were also studied in the presence of EDCs mixtures. Removal rates were different in micropollutant mixtures if compared with removal rates obtained treating individually the different molecules with alternating results towards BPA and NP, respectively. Improvement of enzyme performances in NP removal was achieved through immobilization on glass beads.

These results highlight the influence on the enzymatic degradation efficiency of the ratio between xenobiotic concentration and enzyme affinity. Thus, a challenge still open to face EDCs degradation is the discovery/tailoring enzymes capable of degrading the target compounds with an affinity constant of the same order of magnitude with respect to the actual concentrations of the EDCs in the environment. As a fact, since EDCs concentration in real wastewater is very low (ng/L), enzymes displaying a very high efficiency (high turnover together with high affinity) towards this molecule are excellent candidates to efficiently achieve their removal.

## Figures and Tables

**Table 1:** Degradation of MTPRB and BTPRB in the presence of 200 *μ*M of ABTS, or AS after a 1 h treatment at pH 5.0 25°C with 1.5 U/mL of laccases. All results are averages from two replicate experiments and the standard deviation is less than 10%.

Enzymes	MTPRB		BTPRB	
	(% degradation)		(% degradation)	
	ABTS	AS	ABTS	AS
POXC	—	50	15	60
rPOXA1b	—	35	—	40
1H6C	5	7	7	8
Novoprime Base 268	—	40	—	50

## References

[1] Schug TT, Janesick A, Blumberg B, Heindel JJ (2011). Endocrine disrupting chemicals and disease susceptibility. *Journal of Steroid Biochemistry and Molecular Biology*.

[2] Combalbert S, Hernandez-Raquet G (2010). Occurrence, fate, and biodegradation of estrogens in sewage and manure. *Applied Microbiology and Biotechnology*.

[3] Diamanti-Kandarakis E, Bourguignon J-P, Giudice LC (2009). Endocrine-disrupting chemicals: an Endocrine Society scientific statement. *Endocrine Reviews*.

[4] European Union Commission staff working document. An implementation of the community strategy for endocrine disrupters—a range of substances suspected of interfering with the hormone systems of humans and wildlife.

[5] Liu Z-H, Kanjo Y, Mizutani S (2009). Removal mechanisms for endocrine disrupting compounds (EDCs) in wastewater treatment—physical means, biodegradation, and chemical advanced oxidation: a review. *Science of the Total Environment*.

[6] Galliker P, Hommes G, Schlosser D, Corvini PF-X, Shahgaldian P (2010). Laccase-modified silica nanoparticles efficiently catalyze the transformation of phenolic compounds. *Journal of Colloid and Interface Science*.

[7] Cabana H, Jones JP, Agathos SN (2007). Elimination of endocrine disrupting chemicals using white rot fungi and their lignin modifying enzymes: a review. *Engineering in Life Sciences*.

[8] Yang S, Hai FI, Nghiem LD (2013). Understanding the factors controlling the removal of trace organic contaminants by white-rot fungi and their lignin modifying enzymes: a critical review. *Bioresource Technology*.

[9] Kim Y-J, Nicell JA (2006). Impact of reaction conditions on the laccase-catalyzed conversion of bisphenol A. *Bioresource Technology*.

[10] Cabana H, Jiwan J-LH, Rozenberg R (2007). Elimination of endocrine disrupting chemicals nonylphenol and bisphenol A and personal care product ingredient triclosan using enzyme preparation from the white rot fungus *Coriolopsis polyzona*. *Chemosphere*.

[11] Nicolucci C, Rossi S, Menale C (2011). Biodegradation of bisphenols with immobilized laccase or tyrosinase on polyacrylonitrile beads. *Biodegradation*.

[12] Auriol M, Filali-Meknassi Y, Tyagi RD, Adams CD (2007). Laccase-catalyzed conversion of natural and synthetic hormones from a municipal wastewater. *Water Research*.

[13] Giardina P, Faraco V, Pezzella C, Piscitelli A, Vanhulle S, Sannia G (2010). Laccases: a never-ending story. *Cellular and Molecular Life Sciences*.

[14] Mohapatra DP, Brar SK, Tyagi RD, Surampalli RY (2011). Parameter optimization of ferro-sonication pre-treatment process for degradation of bisphenol A and biodegradation from wastewater sludge using response surface model. *Journal of Hazardous Materials*.

[15] Jie X, Jian Mei L, Zheng F, Gong L, Zhang B, Yu J (2013). Neurotoxic effects of nonylphenol: a review. *The Central European Journal of Medicine*.

[17] Soni MG, Burdock GA, Taylor SL, Greenberg NA (2001). Safety assessment of propyl paraben: a review of the published literature. *Food and Chemical Toxicology*.

[18] Harvey PW, Darbre P (2004). Endocrine disrupters and human health: could oestrogenic chemicals in body care cosmetics adversely affect breast cancer incidence in women? A review of evidence and call for further research. *Journal of Applied Toxicology*.

[19] Tsai MJ, Kuo PL, Ko YC (2012). The association between phthalate exposure and asthma. *Kaohsiung Journal of Medical Sciences*.

[20] Palmieri G, Giardina P, Marzullo L (1993). Stability and activity of a phenol oxidase from the ligninolytic fungus *Pleurotus ostreatus*. *Applied Microbiology and Biotechnology*.

[21] Garzillo AM, Colao MC, Buonocore V (2001). Structural and kinetic characterization of native laccases from *Pleurotus ostreatus*, *Rigidoporus lignosus*, and *Trametes trogii*. *Journal of Protein Chemistry*.

[22] Giardina P, Palmieri G, Scaloni A (1999). Protein and gene structure of a blue laccase from *Pleurotus ostreatus*. *Biochemical Journal*.

[23] Macellaro G, Baratto MC, Piscitelli A (2014). Effective mutations in a high redox potential laccase from *Pleurotus ostreatus*. *Applied Microbiology and Biotechnology*.

[24] Miele A, Giardina P, Notomista E, Piscitelli A, Sannia G, Faraco V (2010). A semi-rational approach to engineering laccase enzymes. *Molecular Biotechnology*.

[25] Gordon CL, Khalaj V, Ram AFJ (2000). Glucoamylase: green fluorescent protein fusions to monitor protein secretion in *Aspergillus niger*. *Microbiology*.

[26] Gassara F, Brar SK, Verma M, Tyagi RD (2013). Bisphenol A degradation in water by ligninolytic enzymes. *Chemosphere*.

[27] Nitheranont T, Watanabe A, Suzuki T, Katayama T, Asada Y (2011). Decolorization of synthetic dyes and biodegradation of bisphenol a by laccase from the edible mushroom, *Grifola frondosa*. *Bioscience, Biotechnology and Biochemistry*.

[28] Kim Y, Yeo S, Kim MK, Choi HT (2008). Removal of estrogenic activity from endocrine-disrupting chemicals by purified laccase of *Phlebia tremellosa*. *FEMS Microbiology Letters*.

[29] Fukuda T, Uchida H, Takashima Y, Uwajima T, Kawabata T, Suzuki M (2001). Degradation of bisphenol A by purified laccase from *Trametes villosa*. *Biochemical and Biophysical Research Communications*.

[30] Bourbonnais R, Paice MG (1990). Oxidation of non-phenolic substrates. An expended role for laccase in lignin biodegradation. *FEBS Letters*.

[31] Cañas AI, Camarero S (2010). Laccases and their natural mediators: biotechnological tools for sustainable eco-friendly processes. *Biotechnology Advances*.

[32] Tsutsumi Y, Haneda T, Nishida T (2001). Removal of estrogenic activities of bisphenol A and nonylphenol by oxidative enzymes from lignin-degrading basidiomycetes. *Chemosphere*.

[33] Mizuno H, Hirai H, Kawai S, Nishida T (2009). Removal of estrogenic activity of iso-butylparaben and n-butylparaben by laccase in the presence of 1-hydroxybenzotriazole. *Biodegradation*.

